# EVALUATING AN INNOVATIVE USE OF CIVIL MONETARY PENALTY FUNDS: THE MA SUPPORTIVE PLANNING AND OPERATIONS TEAM (SPOT)

**DOI:** 10.1093/geroni/igz038.1877

**Published:** 2019-11-08

**Authors:** Rosanna M Bertrand, Gabrielle R Katz, Teresa M Mota, Terry Moore, Jennifer Pettis, Katherine T Fillo, Katherine C Saunders, Chiara S Moore

**Affiliations:** 1 Abt Associates, Inc., Cambridge, Massachusetts, United States; 2 New York University Rory Meyers College of Nursing, Nurses Improving Care for Healthsystem Elders (NICHE), New York, New York, United States; 3 Massachusetts Department of Public Health, Bureau of Health Care Safety & Quality, Boston, Massachusetts, United States

## Abstract

The Office of the Inspector General reported in 2014, that one in three NH residents experienced an adverse event within 35 days of admissions causing lasting or temporary harm. Thus, state departments of public health (DPH) were implored to invest in improving NH quality and safety. Using Civil Monetary Penalty funds, the Massachusetts DPH, developed the SPOT Initiative to innovatively provide NH teams with technical assistance and training to enhance their federally required Quality Assurance & Performance Improvement (QAPI) programs. Selection criteria included NH Compare 5-Star and MA scorecard ratings and geographic spread. To assess program effectiveness, the SPOT Team collected a range of data in each of the three SPOT years (e.g., QAPI assessments, leadership interviews and surveys, and training evaluations). Results demonstrated the success of the Initiative. Assessment data indicated an increase in QAPI readiness in each subsequent year overall and within of the each QAPI assessment domains (Design and Scope; Governance and Leadership; Feedback, Data Systems, and Monitoring/Systematic Analysis; Performance Improvement Projects and Systematic Analysis/Systemic Action). In Year 1, the overall data collected from the assessments demonstrated that 78% of the NHs that engaged with SPOT had “Not Started” or “Just Started” (1.8/5) implementation of the key QAPI measures. By Year 3, only 13% of NH teams rated themselves in these initial categories, whereas, 57% rated themselves as “Almost There” or “Doing Great” (3.92/5). Further, feedback from most SPOT NH teams was extremely positive as evidenced by high evaluation rankings following initiative learning sessions.

